# Scientific

**Published:** 1889-05

**Authors:** 


### SCIENTIFIC ITEMS.

---

**Whistling Speech.**—At the last meeting of the Berlin Anthropological Society, Lieutenant Quedenfeldt, a German officer who has lived on Gomero Island, one of the canary group, described a whistling language which is used by the inhabitants. The language does not consist of any arbitrary series of signals or sounds. It is described as ordinary speech translated into articulate whistling, each syllable having its own appropriate tone. The Gomero uses both fingers and lips when whistling, and Lieutenant Quendenfelt asserts that he can carry on a conversation with a neighbor a mile off, who perfectly understands all he is saying. The practice is confined to Gomero Island, and is quite unknown to the other islands of the archipelago. The adoption of the whistling language is said to be due to the peculiar geographical construction of Gomero Island. It is traversed by numerous gullies and deep ravines, running out in all directions from the central plateau. As they are not bridged they can only be crossed with great difficulty; hence a man living within a stone's throw of another in a straight line has often to go many miles when he wishes to see and speak to his neighbor. This, it is conjectured, led to the adoption of whistling as a useful means of communication, which has gradually assumed the proportions of a true substitute for speech.—*Leonard's Medical Journal.*

**The Gatling Gun.**—This weapon, the first of quick-firing artillery, has an interesting history. In the New York *Commercial Advertiser* Dr. Gatling tells an instructive story of the development and introduction of the gun. The idea of it originated in a conversation Dr. Gatling had in Indianapolis in 1861 with a friend of his, Benjamin Harrison, now president. One of the new guns was shown to General Pipley, then Chief of Army Ordnance, who refused to have anything to do with it, and said the flint lock was the surest and best weapon anyway, as his successor in office practically declares the Springfield breech-loader to be better than the magazine guns with which the rest of the world is arming itself. General Butler gave orders for the eleven guns which had been manufactured, and later Mr. Stanton ordered a trial of them. Dr. Oatling says:

"I went to Fortress Monroe and tested them, and made a great success. The young officers at the fort tried to play a trick on me. At their howitzers they had trained artillerists. To me they assigned three old negroes. I saw through the game, and asked Col. Baylor, who was in command, to give me an hour in which to instruct my men how to use the gun. This he readily assented to, and I began drilling my 'recruits.' They learned very quickly, and in the hour I was ready. The firing was a competitive examination, and with my three old negroes I fired and made about three hits to one on the target to those made by the old guns. Mr. Stanton then gave me an order for

$175,000 worth of the guns. Since I have sold a great many in this country and every country almost on earth. England has them, and so have Turkey, Austria, France, Russia and Italy."—*Scientific American.*

**Peach Stone Fuel.**—It has been demonstrated in Vaca Valley that peach stones will make as good a fire for household purposes as the best kind of coal in the market, says the Valejo (Cal.) *Chronicle.* The fruit growers, instead of as heretofore throwing the pits away, dispose of the stones at the present time at the rate of $6 a ton. A sack of the stones will weigh about 80 pounds and will last as long as an equal number of pounds of coal, and give a greater intensity of heat. At many of the orchards in the valley may be seen great stacks of peach and apricot stones which will eventually find their way to San Francisco and other places to be sold for fuel. The apricot stones do not burn as readily as the peach, and will not command as good a price. The fruit raisers will undoubtedly be pleased to learn that they now have another source of revenue open to them. A large number of peaches are dried during the summer season for shipment. As soon as the owners find that they have a market for the stones, a greater number of pounds will be dried than heretofore.—*Ib.*

**Hypnotism.**—Hypnotism thrives in Washington. Two gentlemen interested in psychological studies, Mr. W. A. Croffut, executive officer of the Geological Survey, and Gov. N. J. Coleman, Commissioner of Agriculture, give occasional *soirees hypnotiques*, at which they hypnotize numbers of "sensitives." During some recent experiments by Mr. Croffut, two young ladies, temporary victims of the hypnotic hallucination, were taken into an imaginary picture gallery and there left, while the operator turned his attention to a young man who was engaged in the dangerous pastime of catching crocodiles. On returning to the ladies, Mr. Croffut found that he could not make them cognizant of his presence. They did not appear to see him, or hear his voice, and when he stood directly in front of them they took no notice of him whatever. It was a new and somewhat alarming experience, and a quarter of an hour passed before the hypnotizer re-established his domination and brought them back from the land of dreams.—*Science.*

**Myrtol.**—Myrtol is a perfectly clear fluid, and represents that constituent of oil of myrrh which boils at 160° to 170° C. Linderm is the only clinician who has instituted any detailed trials with myrtol. Eichhorst himself was first to apply it in gangrene of the lungs. The results which he obtained were so surprisingly favorable that he has used the drug in numerous cases, and is now convinced that, as a disinfectant of the air passages, myrtol has not its equal. The drug is best given in gelatine capsules (à 0·15, prepared in Paris, or by Pohl, of Schonbaum-Danzig). If a capsule is crushed, the peculiar odor clings to the room for a long time, and if a capsule is swallowed the breath emanates the characteristic odor for many hours, and often for a couple of days. In putrid bronchitis and pneumonic gangrene two to three capsules should be taken every two hours to deodorise and disinfect the parts. The effect is rapid, and a few capsules suffice to remove the bad odor, and often to produce a permanent improvement. The drug is, however, apt to cause a temporary loss of appetite. Myrtol has also been tried against tuberculosis, but the drug has proved utterly useless against the baccillus tuberculosis.—*Therapeutic Gazette.*

